# A social learning primacy trend in mate-copying: an experiment in *Drosophila melanogaster*

**DOI:** 10.1098/rsos.240408

**Published:** 2024-06-19

**Authors:** Ricardo Santiago Araújo, Sabine Nöbel, Diogo F. Antunes, Etienne Danchin, Guillaume Isabel

**Affiliations:** ^1^ Laboratoire & Évolution Diversité Biologique (EDB UMR 5174), Université de Toulouse Midi-Pyrénées, CNRS, IRD, UPS, 31062 Toulouse, France; ^2^ Université Toulouse 1 Capitole and Institute for Advanced Study in Toulouse (IAST), Toulouse, France; ^3^ Department of Zoology, Animal Ecology, Martin-Luther-University Halle-Wittenberg, 06120 Halle (Saale), Germany; ^4^ Centre de Recherches sur la Cognition Animale (CRCA), Centre de Biologie Intégrative (CBI), CNRS UMR 5169, Université de Toulouse Midi-Pyrénées, Toulouse, France

**Keywords:** observational learning, social learning, learning plasticity, reversal learning, mate-copying, mate choice

## Abstract

Social learning is learning from the observation of how others interact with the environment. However, in nature, individuals often need to process serial social information and may favour either the most recent information (recency bias), constantly updating knowledge to match the environment, or the information that appeared first in the series (primacy bias), which may slow down adjustment to environmental change. Mate-copying is a widespread form of social learning in a mate choice context related to conformity in mate choice, and where a naive individual develops a preference for a given mate (or mate phenotype) seen being chosen by conspecifics. Mate-copying is documented in most vertebrate taxa and in the fruit fly *Drosophila melanogaster*. Here, we tested experimentally whether female fruit flies show a primacy or a recency bias by presenting pictures of a female copulating with one of two contrastingly coloured male phenotypes. We found that after two sequential contradictory demonstrations, females show a tendency to prefer males of the phenotype preferred in the first demonstration, suggesting that mate-copying in *D. melanogaster* is not based on the most recently observed mating and may be influenced by a form of primacy bias.

## 1. Introduction

Animals can learn about their environment either by themselves (individual or asocial learning, i.e. trial-and-error tactic) or by watching others interact with the environment (social learning) [1,2]. Social learning, which has been documented in a wide variety of species [3–8], enables individuals to quickly respond to environmental change [9,10] while minimizing the costs and risks of individual exploration. For instance, vultures (*Coragyps atratus*) searching for carcasses are drawn to groups of already feeding conspecifics [11], and naive red squirrels (*Tamiasciurus hudsonicus*) learn faster how to open nuts in the presence of experienced individuals [12].

A special form of social learning is mate-copying in which an observer individual is influenced by the mate choice of same-sex conspecifics. Mate choice is one of the major fitness-affecting decisions in the life cycle of sexually reproducing organisms. However, assessing mate quality through mate sampling would be far too costly, especially for unexperienced individuals [13]. Hence, using public information from other individuals’ matings constitutes an effective way to circumvent the costs of individual sampling [14–16]. Mate-copying exists in a variety of vertebrates, as well as in the fruit fly (*Drosophila melanogaster*) (reviewed in [15,16]). In the latter species, evidence comes from a series of experiments involving traits resulting from differential male feeding substrates [17], artificially coloured male phenotypes [17–23], as well as mutants with a different wing shape [24], suggesting significant levels of behavioural flexibility.

Behavioural flexibility refers to the ability to adjust behaviour to environmental variation [25,26]. It can be mediated by social learning strategies [27] involving biases affecting what, when and from whom individuals should learn (i.e. social information transmission biases [28,29]). In a serial paradigm with a temporal dimension (the ‘when’), two biases that may influence behavioural flexibility are the recency and primacy biases. The recency bias, sometimes referred to as the recency effect, is the tendency to favour or more easily recollect recent information [30,31]. It is expected when previously acquired social information becomes outdated so that individuals benefit from updating it. It thus reduces the risks of outdated social information in changing environments [1]. Conversely, a primacy bias is a tendency to favour the information that was acquired first [32]. It makes behaviour more resistant to environmental change and reduces behavioural flexibility. These two biases can interact in some cases [33]. Both biases have been documented, often simultaneously in long series where individuals favour the first and the last observations in comparison to those in the middle, in humans [31,34] and other species [35–40]. Although recency and primacy biases should influence behavioural flexibility, their role in mate-copying remains unexplored.

Here, we experimentally studied the role of the recency and primacy biases in mate-copying in the fruit fly (*D. melanogaster*) by providing them with two opposite pieces of information in sequence. We use a new, more controlled protocol in which demonstrations consist of flat pictures of a female copulating with one of two males with contrasting phenotypes, followed by a mate choice test [23]. We could thus test whether observer females gave more weight to the first demonstration (primacy bias) or the second one (recency bias).

## 2. Methods

### 2.1. Fly maintenance

We used *D. melanogaster* of the laboratory strain Canton-S raised in 30 ml vials containing 8 ml medium of corn flour, agar and dry yeast. Rearing and experimental conditions were kept constant at $25\pm 1^{\circ }$ C and 55±5 % humidity with a 12 : 12 h light : dark cycle. Newly emerged flies were collected without anaesthesia by gentle aspiration within 6 h of emergence from tubes without mature adults and separated by sex to ensure they remained virgin and naive about mate choice. Experiments were carried out exclusively with 3- or 4-day-old virgin flies. Right before mate choice experiments, virgin males were randomly powdered with artificial green (Shannon Luminous Materials, Inc., B-731) or pink (BioQuip Products, Inc., 1162R) powders, and left in food vials for at least 20 min to remove excess powder. Flies were used only once.

### 2.2. Behavioural trials

**Figure 1.**
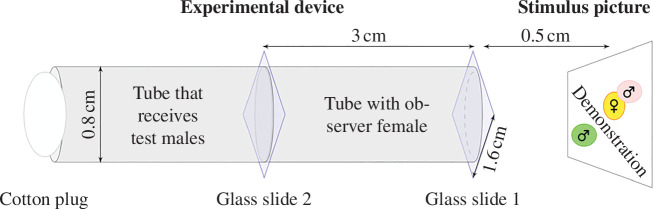
The experimental device. The device used in mate-copying experiments consists of two adjacent tubes separated by a see-through glass slide. The observer female is placed in the tube on the right, which is closed by another glass slide in front of a picture of a female copulating with one of two males. After the demonstrations, the picture is covered and males are introduced through the left-side tube. The mate choice test starts when the slide partitioning of the two tubes is removed, and the flies are allowed to interact.

**Figure 2.**
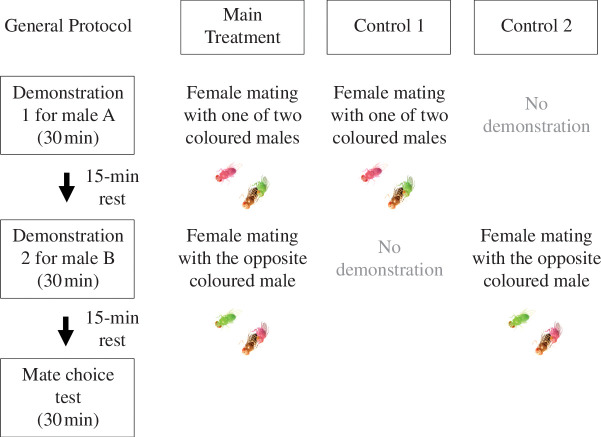
The three experimental treatments. All three treatments lasted for a total of 2 h divided into two 30 min demonstration periods, one 30 min mate choice test and two 15 min breaks between demonstrations and before the mate choice test that lasted for 30 min. Male colours used for the demonstrations were balanced between trials.

A mate-copying experiment in *D. melanogaster* consists of two distinct phases. First, a demonstration phase where the observer female can acquire social information from demonstrators, followed by a mate choice test of the observer. We used the same set-up as previous studies (figure 1) [23]. It was made of two adjacent plastic tubes (0.8 cm × 3 cm each) that were plugged on one side with cotton, and on the other by a microscopy cover slide (1.6 cm × 1.6 cm) that served as a window from which the observer female could watch the demonstration. Another similar glass partition was placed between the two tubes, separating them. The observer female was placed in the tube adjacent to the side of the demonstration. Demonstrations consisted of one life-sized picture of a female mating with one of the coloured males with a male of the other colour standing by, as previously done by Nöbel *et al*. [23]. Pictures were approximately 0.5 cm from the focal female tube. We used 26 different pictures to reduce pseudo-replication, and we found that the picture ID did not explain the variation in our data. Each trial consisted of two 30 min demonstrations in two successive slots (demonstration slots 1 and 2) followed by a mate choice test (see figure 2). Demonstrations were followed by a 15 min resting time during which the observer female remained in the experimental device. Treatments consisted of a main treatment and two controls (figure 2) and ended with a 30 min mate choice test, where the observer female had the choice between a pink and a green living male. These males were initially added to the tube separated by the glass partition after the demonstrations were finished, which was removed at the start of the mate choice test.

The main treatment consisted of two sequential demonstrations providing conflicting social information (i.e. the demonstrator female copulated with the male of one colour in the first demonstration and with the male of the other colour in the second; figure 2). To control for the time gap between each demonstration and the mate choice test, we replicated that protocol in the controls but provided no visual stimulus in the second (‘Control 1’) or first demonstration (‘Control 2’). These controls also tested whether flies were learning socially with the current protocol.

### 2.3. Trial recording and analysis

All mate choice tests were video recorded in 4 k resolution, 24 FPS using Nikon Z50 cameras equipped with 16–50 mm/3.5–6.3 lenses suspended 40 cm over the experimental devices to minimize disturbances. Behavioural analysis was performed using the media player software PotPlayer (Kakao Corp., version 1.7.21631). To minimize a potential observer bias, video recording and analysis were double-blind as they were performed days or weeks after the experiment and R.S.A. did not know the actual treatment of each trial while collecting the behavioural data from the videos. For each trial, we recorded the colour of the male(s) that courted the observer female and the colour of the male chosen for copulation. We also recorded the date, time of the day, room humidity, room temperature and air pressure at the start of each trial.

### 2.4. Mate-copying index

As in previous studies, we only kept trials in which copulation occurred after both males courted the female during the mate choice test (205 out of 462 trials in total), because this was the only context in which we could be sure that the observer female was in a position to choose [19]. For each trial, we assigned a mate-copying score of 1 if the observer female’s choice matched the colour with which the demonstrator female mated during the last demonstration, and 0 when it did not. The last demonstration was the second demonstration in the treatment and the unique demonstration in both controls. A mate-copying index (MCI) was calculated as the average of the mate-copying scores per treatment for the trials in which copulation occurred after both males courted the female.


$$MCI=\frac{Total_{females\;that\;copied\;focal\;demo}}{Total_{mate\;choice\;trials}}.$$

### 2.5. Statistical analysis

Data analyses were performed with version 4.2.1 of R software (R Core Team, 2022). The significance of the departure from random choice was tested with a binomial test. The confidence interval of the binomial proportion at each treatment was calculated with the Agresti–Coull method (DescTools package [41]). To compare treatments, we used generalized linear mixed models (GLMMs) with binary logistic regression (package lme4). We used Wald chi-square tests implemented in the ANOVA function of the car package [42] to test the significance of fixed effects. The starting GLMMs had MCI scores as the dependent variable, and treatment, air pressure, humidity and temperature as fixed effects and block (i.e. group of trials (10–16) performed simultaneously) as a random effect. Model selection was performed using backward selection, resulting in the exclusion of air pressure, humidity and temperature as they never had a significant effect.

## 3. Results

**Figure 3.**
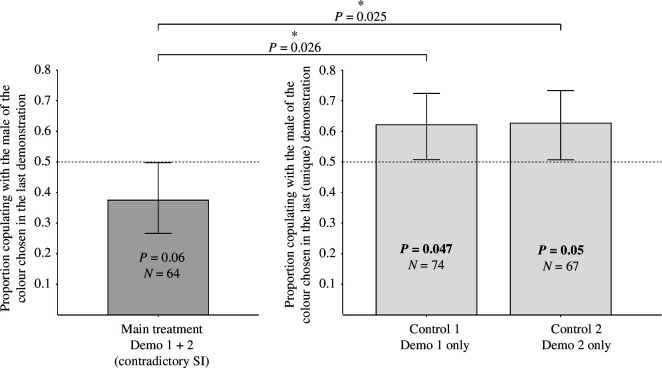
Mate-copying indices (MCIs) of the three treatments performed in parallel. Observer females in the main treatment received two demonstrations in successive time slots providing contradictory social information (SI); those of control 1 and control 2 only received information in the first or second demonstration slot, respectively. The MCI is the fraction of females that copied the last demonstration. Dashed line: random choice. Numbers in bars: sample sizes. *p*-values in bars: *p*-value of the test of departure from random choice (binomial tests). *p*-value above the horizontal bar: GLMM comparing the three treatments. Confidence intervals were calculated with the Agresti–Coul method.

Observer females in the main treatment, who received contradictory social information, showed a trend towards a preference for the male phenotype of the first demonstration (binomial test, *n* = 64, *p* = 0.06, left bar of figure 3). Observer females that received only one demonstration (Controls 1 and 2) copied the choice of that single demonstration (Control 1: binomial test, *n* = 74, *p* = 0.047; Control 2: binomial test, *n* = 67, *p* = 0.05, middle and right bars of figure 3). The main treatment was significantly different from Control 1 (GLMMs, *p* = 0.026) and Control 2 (GLMMs, *p* = 0.025).

## 4. Discussion

Our results suggest that female fruit flies receiving two sequential demonstrations providing contradictory information tend to copy the first rather than the second demonstration (main treatment), suggesting a form of primacy bias that seems to contradict the general wisdom that learners should prioritize newer social information [1]. Observer females that received a single demonstration, whether early (Control 1) or late (Control 2), copied their conspecifics, which is consistent with the result of previous studies [17–22].

These results suggest that *D. melanogaster* does not copy the most recently observed choice and may have a primacy bias regarding their socially learned preference for male phenotypes. Such a primacy bias is reminiscent of early life imprinting [43], in which the first observation is critical to the animal’s development, thus becoming ingrained [44]. There are multiple examples in which the first contact in a critical period, usually with a parent, somehow determines the behavioural phenotype of the individual, with examples of sexual imprinting across many animal taxa, including mammals [45], birds [46], fish [47], insects [48] and spiders [49]. In the current study, observer females initially were naive in respect to male quality and reproduction. Consequently, they might have been in a state of higher susceptibility to such social information similar to the sensitive period of imprinting. Hence, our result may reveal an ‘imprinting-like’ phenomenon in the fruit fly, the function of which may be analogous to early-in-life imprinting. The evolutionary advantage of a primacy bias in *D. melanogaster* may be its simplicity. While a more sophisticated learning system that would constantly update to match newer information would allow for greater behavioural flexibility, it would also presumably be more energetically costly to maintain, requiring constant surveillance of the social environment.

This raises questions about the underlying mechanisms and evolutionary origin of mate-copying in the fruit fly. Although our two male phenotypes are artificial, drastic eye or body colour changes following a single mutation have been documented in *Drosophila*, implying that the existence of such contrasted phenotypes is not totally implausible in nature. Structural wing colour patterns in the transparent wings [50] can be detected by colour vision in insects and produce contrasting phenotypes. Furthermore, we also found the existence of mate-copying using genetically affected phenotypes [24], suggesting the generality of this phenomenon. Interestingly, flies are able to detect subtle differences, such as the presence or absence of eyes, wings or legs, as we have discovered through manipulating fly pictures [23]. This allows us to speculate about the origin of this tendency towards primacy. It may well be that adult fruit flies are so short-lived in nature that most of them have only one breeding opportunity, thus suppressing the selection for a complex updating system. This would also explain the apparent contradiction between a trend towards primacy and the strong conformity in mate-copying found in *Drosophila* [18]. Conformity would predict a recency bias to allow dispersing individuals to adopt local preferences [16], but if individuals are too short-lived to ever disperse, a primacy bias would not be costly.

Experiments with strains other than Canton-S are needed to assess these ideas and the generality of mate-copying in *Drosophila*. Likewise, it will be important to develop protocols that use naturally occurring phenotypes. Nonetheless, the use of contrasting artificial phenotypes avoids statistical fitness differences between male phenotypes and has been particularly efficient in studying the underlying cognitive processes [22,23], thus providing a new animal model to study the neurobiology of social learning [21,51].

Learning from contradictory sequential information can be related to reversal learning. We have previously found that mushroom body (MB) neurons are required for social learning [51]. MBs, composed of Kenyon cells, are modulated by a pair of GABAergic neurons, the inhibitory anterior paired lateral (APL) neurons, which are not involved in visual associative learning, although their activity is required for reversal learning [52]. *Drosophila* therefore have all the neurobiological capabilities needed for successful reversal learning. However, in this experiment, the *Drosophila* did not reverse their choice tendency after the second contradictory demonstration. If the APL neuron pair was also involved in this process, it is conceivable that its activity is not sufficiently solicited during the second demonstration to reverse the initial learning, or that the Kenyon cells involved in the initial learning are so active that they are difficult for the APL neurons to modulate. Multiple reversed demonstrations might reduce the tendency for a primacy bias and even eventually reverse it. Our experiment with two demonstrations does not allow us to test this, but these hypotheses open a tantalizing avenue for the further study of the neurobiology underpinning animal culture.

In conclusion, our results may reveal a limitation of the otherwise unexpectedly well-developed social learning capacities of *D. melanogaster*. While mate-copying provides a certain degree of behavioural flexibility in mate choice in the fruit fly, a primacy bias may constrain that flexibility, which raises intriguing questions about that form of social learning. While social learning has been shown to be widespread in nature, we can expect social learning strategies to vary dramatically depending on the species’ ecology, life history and behavioural context. The coexistence of various learning strategies can sometimes generate conflicts among them (e.g. between familiarity and success biases), with natural selection weighing them differently according to the context. All these questions open the way to further studies on the evolution of mate-copying and social learning in general.

## Data Availability

Data are available on Dryad [53]. Supplementary material is available online [54].
